# Phase II, randomized, open-label study of durvalumab (MEDI4736) or tremelimumab monotherapy, or durvalumab + tremelimumab, in patients with recurrent or metastatic (R/M) squamous cell carcinoma of the head and neck (SCCHN): CONDOR

**DOI:** 10.1186/2051-1426-3-S2-P152

**Published:** 2015-11-04

**Authors:** Jill Gilbert, Christophe Le Tourneau, Hisham Mehanna, Jerome Fayette, Trishna Goswami, Ugochi Emeribe, Anthony Jarkowski, Giovanni Melillo, Lilian L Siu

**Affiliations:** 1 Vanderbilt University, Nashville, TN, USA; 2 Department of Medical Oncology, Institut Curie, Paris & Saint-Cloud, France; 3 Institute of Head and Neck Studies and Education, University of Birmingham, Birmingham, UK; 4 Centre Léon Bérard, University of Lyon, Lyon, France; 5 AstraZeneca, Gaithersburg, MD, USA; 6 Princess Margaret Cancer Centre, Toronto, ON, Canada

## Background

First-line palliative treatment for patients with R/M SCCHN includes platinum-based chemotherapy. There are no standard second-line options upon relapse and median survival is limited. In SCCHN, tumors create a highly immunosuppressive environment and evade immune detection by exploiting inhibitory immune checkpoints such as the programmed cell death ligand-1 (PD-L1)/programmed cell death-1 (PD-1) axis, making immunotherapy an attractive option to study in this disease. Durvalumab (MEDI4736) is a selective, high affinity human IgG1 mAb that blocks PD-L1 binding to PD-1 (IC_50_ 0.1 nM) and CD80 (IC_50_ 0.04 nM) that has shown promising antitumor activity in the SCCHN cohort of a Phase I/II study (NCT01693562). Tremelimumab is a selective human IgG2 mAb inhibitor of cytotoxic T-lymphocyte-associated antigen-4 (CTLA-4) that has been combined with durvalumab in a Phase Ib study in patients with NSCLC (NCT02000947), with encouraging clinical activity and a manageable safety profile. While anti-PD-1/PD-L1 monotherapy may be associated with greater clinical benefit in patients with PD-L1^+^ tumors, combination therapy has clinical activity in both PD-L1^+^ and PD-L1^–^ patients in several solid tumors, enhancing the antitumor activity of anti-PD-1/PD-L1 agents in patients with PD-L1^–^ tumors. The PD-L1 and CTLA-4 pathways are non-redundant and preclinical data indicate targeting both may induce synergistic antitumor effects. Here we describe the CONDOR study (NCT02319044), a Phase II study to determine the efficacy and safety of durvalumab monotherapy, tremelimumab monotherapy, and their combination in PD-L1^–^ R/M SCCHN patients who have failed prior platinumtherapy.

## Methods

**Figure 1:**
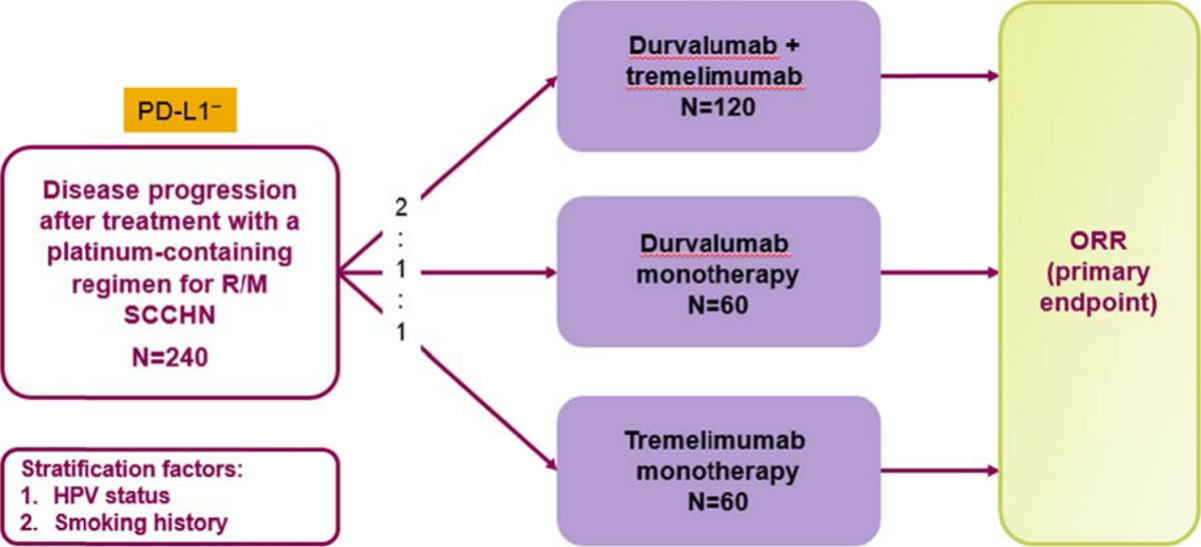


This is a randomized, open-label, multicenter, global, Phase II study in immunotherapy-naïve patients with PD-L1^–^ R/M SCCHN who have progressed during or after treatment with a platinum-containing regimen for R/M disease. Patients with tumoral PD-L1 expression below a pre-specified cut-off level of < 25% tumor cells with membrane staining, as determined by an immunohistochemistry assay, are deemed PD-L1^–^. Patients (N=240) will be randomized 1:1:2 to receive durvalumab (10 mg/kg IV) monotherapy; tremelimumab (10 mg/kg IV) monotherapy; or durvalumab (20 mg/kg IV) plus tremelimumab (1 mg/kg IV) combination therapy for up to 12 months (Figure 1). Stratification factors include human papillomavirus (HPV) and smoking history. The primary endpoint is objective response rate (ORR; RECIST v1.1), based on independent central review. Secondary endpoints will further assess disease control rate, duration of response, progression-free survival, and overall survival; safety (CTCAE v4.03) and tolerability; and health-related quality of life. Exploratory outcomes include pharmacokinetics, immunogenicity, and potential biomarkers of response to treatment. Recruitment is ongoing.

## Trial registration

ClinicalTrials.gov identifier NCT02319044.

